# *Akkermansia muciniphila* administration ameliorates streptozotocin‐induced hyperglycemia and muscle atrophy by promoting IGF2 secretion from mouse intestine

**DOI:** 10.1002/imt2.237

**Published:** 2024-10-01

**Authors:** Chi Zhang, Zhihong Wang, Xu Liu, Xiangpeng Liu, Tong Liu, Yu Feng, Zhengrong Yuan, Zhihao Jia, Yong Zhang

**Affiliations:** ^1^ Cambridge‐Suda Genomic Resource Center, Suzhou Medical College Soochow University Suzhou China; ^2^ Jiangsu Key Laboratory of Neuropsychiatric Diseases Research Soochow University Suzhou China; ^3^ Institute of Pain Medicine and Special Environmental Medicine Nantong University Nantong China; ^4^ Department of Endocrinology The Second Affiliated Hospital of Soochow University Suzhou China; ^5^ College of Biological Sciences and Technology Beijing Forestry University Beijing China

## Abstract

Type 1 diabetes mellitus (T1DM) is an autoimmune disease that can lead to severe diabetic complications. While the changes and correlations between gut microbiota and the pathogenesis of T1DM have been extensively studied, little is known about the benefits of interventions on gut bacterial communities, particularly using probiotics, for this disease. In the present study, we reported that the mice surviving after 5 months of streptozotocin (STZ) injection had reduced blood glucose level and recovered gut microbiota with increased *Akkermansia muciniphila* proportion. Gavage of heat‐killed *A. muciniphila* increases the diversity of gut microbiota and elevated immune and metabolic signaling pathways in the intestine. Mechanistically, *A. muciniphila* treatment promoted the secretion of insulin‐like growth factor 2 (IGF2) which subsequently activated IGF2 signaling in skeletal muscles and enhanced muscle and global metabolism. Our results suggest that the administration of heat‐killed *A. muciniphila* could be a potential therapeutic strategy for T1DM and its associated hyperglycemia.

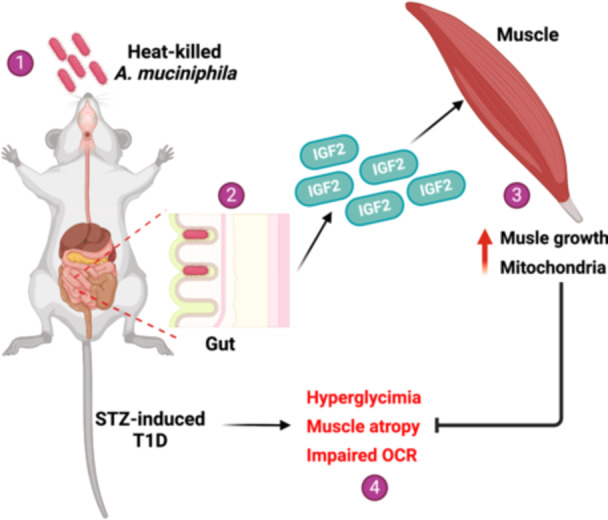

Type 1 diabetes mellitus (T1DM) characterized by the destruction of pancreatic β‐cells, constitutes approximately 10%–15% of all DM cases, and is most commonly diagnosed in childhood diabetes [1]. The pathogenesis of T1DM is associated with the formation of T1DM‐associated autoantibodies, stemming from cognate interactions between T cells and B cells [2]. Complications of T1DM arise from prolonged hyperglycemia, making the management of T1DM primarily focused on insulin‐related therapy for glycemic control [1]. Complementing insulin therapy, lifestyle modifications such as dietary choices and regular exercise are essential for successful T1DM management. Despite these interventions, a significant number of T1DM patients continue to experience diabetic complications, emphasizing the urgent need for research efforts in the development of new treatments.

Large‐scale epidemiological studies have explored the changes and potential roles of gut microbiota in the pathogenesis of T1DM. Nonobese diabetic (NOD) mice which lacking *MyD88*, exhibit altered gut microbiota in the distal gastrointestinal tract and are protected from developing DM through germ‐free administration of pathogen‐free microbiota [3]. In children with preclinical T1DM, gut microbiota exhibits an increased Bacteroidetes/Firmicutes ratio and decreased diversity [4]. However, whether microbiota changes are a consequence or cause of T1DM remains uncertain and requires further clarification.

*Akkermansia muciniphila* is a prevalent mucin‐degrading Gram‐negative bacterium, constituting approximately 3%–5% of the gut microbiota biomass in humans. Recent studies have highlighted the positive effects of *A. muciniphila* on host metabolism and immunity [5, 6]. The abundance of *A. muciniphila* is correlated with various metabolic disorders [7], particularly obesity and type 2 diabetes mellitus (T2DM). Supplementation with live or heat‐killed *A. muciniphila* ameliorates metabolic endotoxemia, improves gut‐barrier function, and subsequently reverses systemic metabolic defects [8]. However, the potential relationship of *A. muciniphila* with T1DM remains less understood and controversial. In our present study, we observed a significant increase in the abundance of *A. muciniphila* in mice surviving after a 5‐month induction of streptozotocin (STZ)‐induced T1DM, coupled with improved hyperglycemia. Consequently, we employed *A. muciniphila* gavage to investigate the physiological roles and molecular mechanisms of *A. muciniphila* in the progression of T1DM.

## RESULTS

### Mice that survived after 5 months of STZ injection had increased *A. muciniphila*

**Figure 1:**
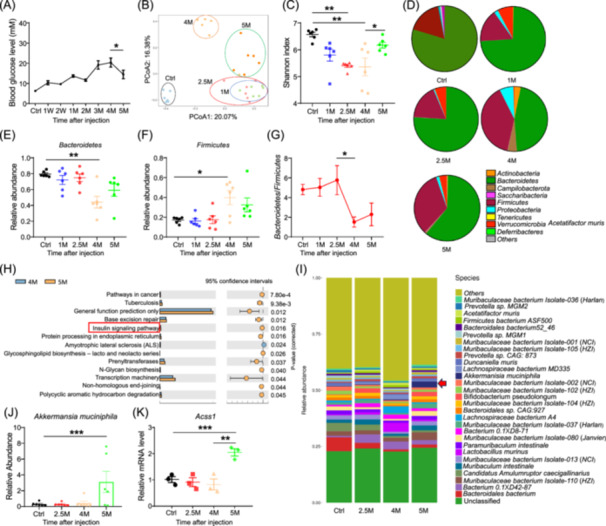
Mice survived after long‐term streptozotocin (STZ) injection has decreased glucose level, altered intestinal microbiota, and increased *Akkermansia muciniphila*. (A) Fasting blood glucose levels of mice before and after 1 week, 2 weeks, 1 months, 2 months, 3 months, 4 months, and 5 months of STZ injection. (B) Principal component analysis (PCA) analysis of 16s rRNA sequencing data of cecal contents from mice after 1, 2.5, 4, and 5 months of STZ injection, *n* = 6. (C) Shannon index of the 16s rRNA sequencing data, *n* = 6. (D) Bacterial community structure analysis at phyla level, *n* = 6. (E–G) Relative abundances of Bacteroidetes (E) and Firmicutes (F), and the ratio of Bacteroidetes/Firmicutes at 1, 2.5, 4, and 5 months after STZ injection, *n* = 6. (H) KEGG pathway analysis of bacterial communities between 4 and 5 months after STZ injection, *n* = 6. (I) Bacterial community structure analysis at phyla level, *n* = 6. Red arrow indicated *A. muciniphila*. (J) Relative abundances of *A. muciniphila* at different times post‐STZ injection, *n* = 6. (K) qRT‐PCR detection of *Acss1* at 2.5‐, 4‐, and 5‐month post‐STZ injection. Data represent mean ± SEM (*t* test: * *p* < 0.05, **p < 0.01, ***p < 0.001). PCA, principal component analysis; PCoA, principal coordinate analysis; qRT‐PCR, quantitative reverse transcription polymerase chain reaction; rRNA, ribosomal RNA; SEM, scanning electron microscopy.

We subjected mice to a single dose of 100 mg/kg STZ injection and kept them until 5‐month postinjection (mpi). The fasting glucose levels of the mice kept increasing after STZ injection and peaked at 4 mpi (Figure 1A), while significantly decreased at 5 mpi compared with those of 4 mpi (Figure 1A). Principal component analysis of 16s rRNA sequencing from cecal contents revealed a clear separation of STZ‐injected groups with the control group (Figure 1B), and 4 mpi was isolated from all other groups (Figure 1B). The Shannon index indicated bacterial community diversities were significantly decreased at 2.5 and 4 mpi, and partially recovered at 5 mpi (Figure 1C). Bacterial community structure as well as the relative proportions of Bacteroidetes and Firmicutes, exhibited significant changes at 4 mpi (Figure 1D–F and Table S1). The ratio of Bacteroidetes/Firmicutes, which was an indicator of the healthiness of the gut, was also significantly decreased at 4 mpi (Figure 1G). The relative proportions of Bacteroidetes and Firmicutes, and the ratio of Bacteroidetes/Firmicutes, were all recovered at 5 mpi (Figure 1D–G). Functional differences of bacterial communities showed minor changes at 1 and 2.5 mpi (Figure S1A,B), expanded at 4 mpi, including T1DM (Figure S1C). While at 5 mpi, bacterial communities involved in the insulin signaling pathway were found upregulated (Figure 1H).

We next performed metagenomic next‐generation sequencing (Table S2) to identify key bacteria species. The results showed that *A. muciniphila* was in the top fold‐changed bacteria species between 4 and 5 mpi (Figure 1I,J). As *A. muciniphila* is involved in the production of short‐chain fatty acids (SCFAs) [9], we analyzed the expression level of SCFA metabolism‐related genes. The mRNA levels *Acss1*, *Ffar2* and *Ffar3* were significantly upregulated at 5 mpi from the intestine (Figures 1K and S1D). These data suggest that the increased glucose levels and disrupted bacterial communities after STZ‐induced T1D are recovered in mice that survived at 5 mpi, with an increased abundance of *A. muciniphila* and enhanced SCFA metabolism.

### *A. muciniphila* gavage protects mice from STZ‐induced muscle atrophy by promoting intestinal insulin‐like growth factor 2 (IGF2) secretion

**Figure 2:**
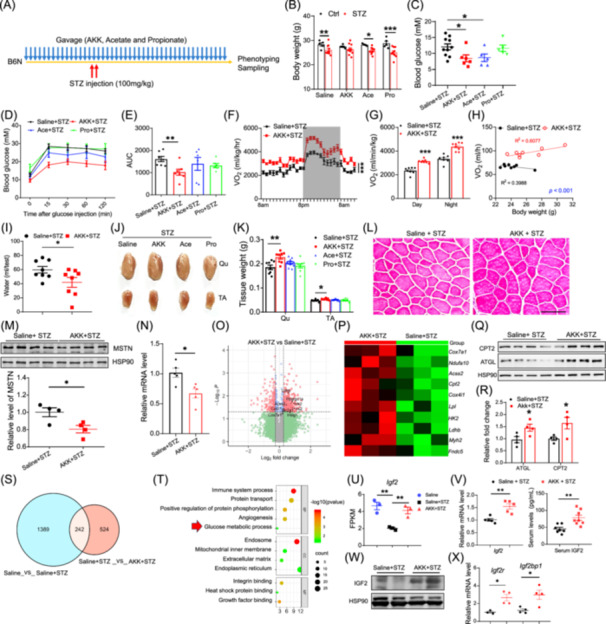
*Akkermansia muciniphila* gavage protects mice from STZ‐induced muscle atrophy by promoting intestinal IGF2 secretion. (A) A scheme showing the gavage and STZ injection on wild‐type C57BL/6J mice. (B) Body weight of mice received gavage of saline, *A. muciniphila* (AKK), sodium acetate (Ace), and sodium propionate (Pro) after 5 weeks of STZ‐induced T1D, *n* = 5 and 10 of mice with/without STZ injection. (C) Fasting glucose levels of mice after 5 weeks of STZ injection, *n* = 10, 6, 5 and 5 for each group, respectively. (D) Blood glucose levels during glucose tolerance test (GTT) on mice after STZ injection. (E) Area under curve (AUC) calculated from GTT. (F–H) O_2_ consumption (F), average day and night O_2_ consumption (G), and correlation between O_2_ consumption and body weight (H) from AKK and saline groups, *n* = 8. (I) Water consumption of mice from AKK and saline groups after STZ injection, *n* = 8. (J, K) Representative images (J) and weights (K) of Quadriceps (Qu) and tibialis anterior (TA) muscles isolated from mice received gavage of saline, AKK, Ace, and Pro after STZ‐induced T1D. (L) Representative H&E staining image of muscle cross‐section from saline and AKK groups. (M) Western blot and calculation of MSTN protein expression level in TA muscles, *n* = 4. (N) Relative mRNA levels of *Mstn* TA muscles from saline and AKK groups after STZ injection, *n* = 5. (O) Volcano plot showing the Log_2_ fold change and −Log_10_ *P* of all genes by RNA sequencing using TA muscles from AKK and saline groups, red dots represented significant differential gene expression (DEGs) with fold change >1.3 while green dots represented unchanged genes. (P) Heatmap of key DEGs involved in lipid and glucose metabolism from AKK and saline groups after STZ injection, *n* = 3. (Q, R) Western blot (Q) and calculation (R) of CPT2 and ATGL protein expression levels in TA muscles, *n* = 4. (S) Venn diagram of overlapping DEGs from untreated versus saline and saline versus AKK. (T) Gene ontology enrichment of all overlapping DEGs indicating the specific changes in glucose metabolic process, *n* = 3. (U) Gene expression levels of *Igf2* from untreated, saline and AKK groups, *n* = 3. (V) qRT‐PCR validation of *Igf2* expression from intestine and serum concentrations of IGF2 protein of saline and AKK groups after STZ injection, *n* = 5 and 8, respectively. (W) Western blot of IGF2 from the intestine of AKK and saline groups after STZ injection. (X) Relative mRNA levels of *Igf2r* and *Igf2bp1* in TA muscles from saline and AKK groups after STZ injection, *n* = 4. Data represent mean ± SEM (*t* test, two‐way ANOVA and ANCOVA: *p < 0.05; **p < 0.01; ***p < 0.001). ANCOVA, analysis of covariance; ANOVA, analysis of variance; ATGL, adipose triglyceride lipase; CPT2, carnitine palmitoyl transferase 2; Ctrl, control; FPKM, fragments per kilobase of transcript per million mapped reads; H&E, hematoxylin and eosin; IGF2, insulin‐like growth factor 2; MSTN, myostatin; qRT‐PCR, quantitative reverse transcription polymerase chain reaction; rRNA, ribosomal RNA; SEM, scanning electron microscopy; STZ, streptozotocin; T1D, type 1 diabetes.

Wild‐type C57BL/6J mice were then orally administered for a month before STZ injection with saline, heat‐killed *A. muciniphila* (AKK), sodium acetate (Ace), and sodium propionate (Pro), respectively (Figure 2A). Mice without STZ injection at the same time were set as control (Ctrl). Then the successful modeling mice continued to receive the same gavage treatment (Figure 2A). At 3‐ and 5‐week post‐STZ injection, body weights of mice from saline, Ace and Pro groups were significantly reduced compared with Ctrl groups, while there was no difference in those of the AKK group (Figures 2B and S2A). The fasting glucose levels of the mice from the AKK and Ace groups were significantly reduced compared with the saline group (Figure 2C). In the glucose tolerance test, mice from the AKK group showed reduced glucose levels and improved glucose sensitivity (Figures 2D,E and S2B). Metabolic analysis showed that *A. muciniphila* gavage significantly elevates the O_2_ consumption, CO_2_ production and Heat production of the mice (Figures 2F–H and S3A–E). While there was no difference in the respiration exchange rate (Figure S3F). *A. muciniphila* gavage also reduced the water intake of STZ‐induced T1D mice, showing improved diabetes syndrome (Figure 2I). While food intake and activity of the mice were not changed (Figure S3G–I).

MRI scanning results showed that mice receiving *A. muciniphila* gavage showed a slight increase in lean mass (Figure S4A). Indeed, the weight of different muscle tissues, including tibialis anterior (TA) and quadriceps, was all larger in mice of the AKK group compared with those of the saline group (Figure 2J,K). Cross‐section of TA also revealed that average fiber size (Figure 2L) and proportions of larger‐sized fibers of the AKK group were significantly increased (Figure S4B,C). Protein levels of myostatin (MSTN), as well as the mRNA levels of *Atrogin‐1*, *Trim63*, and *Mstn*, were all significantly reduced from TA of AKK treated mice than saline group (Figures 2M,N and S4D,E). In addition, weights of different fat depots were not changed between AKK and saline groups (Figure S4F,G).

By high‐throughput RNA sequencing, we identified 1075 and 806 differential expressed genes (DEGs) between control versus saline groups and AKK versus saline groups, respectively (Figures 2O and S5A, and Table S3). KEGG revealed that mitochondrion and insulin receptor binding‐related genes were specifically enriched from those upregulated genes from the AKK group (Figure S5B). The expression levels of *Cpt2*, *Ppargc1α*, *Cox7a1*, *Acss2*, *Hk2*, *Ldhb*, *Slc2a1*, *Irs2*, *Insig1, Fndc5* (*Irisin*, a muscle‐secreted myokine that promotes global metabolism) and *Myh2* (Myosin Heavy Chain 2, key structural protein of muscle) were found upregulated genes after *A. muciniphila* gavage (Figure 2P). In addition, protein levels of adipose triglyceride lipase (ATGL) and carnitine palmitoyl transferase 2 (CPT2) were significantly increased in TA muscle from the AKK group (Figure 2Q,R).

We next investigated the gut microbiota and transcriptome changes, as multiple studies have highlighted the impact of *A. muciniphila* gavage on gut. A 16s rRNA sequencing indicated significantly increased diversity of gut microbiota after *A. muciniphila* gavage (Figure S6 and Table S4). Transcriptome analysis suggested that *A. muciniphila* gavage upregulated genes involved in innate immune response, lipid transport, glucose metabolic process, plasma membrane and carbohydrate binding (Figure S7A–C and Table S5). Western‐blot analysis revealed that protein levels of ATGL, CPT2, and MTCO1 (represented mitochondrial complex 4) were all significantly increased from the intestines of the AKK group (Figure S7C,D).

To identify the key secret proteins that were responsible for *A. muciniphila* treatment‐induced muscle mass, we extracted the DEGs under the same threshold from saline versus control and AKK versus saline, respectively (Figure 2S and Table S6). Pathway enrichment indicated that the 242 overlapped DEGs were mostly involved in the immune system process, endosome and endoplasmic reticulum (Figures 2T and S8A). Particularly, *Igf2*, which encodes a cytokine IGF2 that is critical for skeletal muscle growth and metabolism, was downregulated from the intestine after STZ injection and recovered in the AKK group (Figure 2U). We verified that both mRNA and protein levels of Igf2 from the intestine and serum IGF2 levels were increased after *A. muciniphila* treatment (Figure 2V,W). The protein level of IGF2 was not changed in the TA muscle (Figure S8B). Instead, mRNA levels of *Igf2r* and *Igf2bp1*, which are key elements of IGF2 signaling pathway, were significantly increased in the TA muscle of the AKK group (Figure 2X). These results demonstrate that *A. muciniphila* gavage prevents STZ‐induced atrophy and enhances skeletal muscle metabolism by promoting the secretion of IGF2 from the intestine.

## DISCUSSION

Gut microbiota plays a vital role in host immunity and metabolism, yet studies on the relationship between gut microbiota and T1DM are still in their infancy. We found that spontaneous microbiota recovery may be a driver of improved blood glucose in long‐term surviving T1DM mice. Metagenomics analyses from Finnish children with T1DM have also shown significantly reduced proportions of butyrate‐producing and mucin‐degrading bacteria [10]. In NOD mice, the abundance of *A. muciniphila* is inversely correlated to the risk of developing T1DM [11]. *A. muciniphila* facilitates SCFA production to exert its anti‐inflammatory effects in the intestine by activation of free fatty acid receptor 2 (FFAR2) [12]. We identified that the proportion of *A. muciniphila* and the expression of *Ffar2*, were significantly increased with improved hyperglycemia at 5 mpi. Thus, we could not exclude the possibilities that the antihyperglycemia effect came from *A. muciniphila* or *A. muciniphila*‐mediated SCFA production. To dissect these possibilities, we utilized heat‐killed *A. muciniphila* paralleled with acetate and propionate, respectively. The results indicated that heat‐killed *A. muciniphila*, but not its SCFAs, sufficiently modulate intestinal inflammation and metabolic benefits.

The molecular mechanisms of *A. muciniphila* in combating various diseases have been widely investigated. In addition to live *A. muciniphila*, pasteurized *A. muciniphila* shows significant antidiabetic effects in mice [13], which highlights the direct interaction between *A. muciniphila* and gut barrier. Similar causal evidence was found that heat‐killed *A. muciniphila* could ameliorate STZ‐induced T1DM through the intestine–IGF2–muscle axis. IGF2 and its binding protein IGF2BP1, are critical regulators of muscle differentiation, growth, and function [14, 15]. Thus, we hypothesized that *A. muciniphila* promoted the secretion of IGF2 from the mouse intestine in a direct binding‐dependent manner. Supporting this, IGF2 could be secreted by intestinal epithelial cells [16]. However, rescue or neutralization assays were warranted to confirm the current mechanism. In addition, a significant reduction in blood glucose levels was observed in acetate gavage mice, which is one of the most important metabolites of *A. muciniphila* [7]. Thus, it could not bypass the metabolic and secretory roles of *A. muciniphila*. Token together, the metabolic benefits of *A. muciniphila* should be a combination of improved gut microbiota and gut‐barrier functions, through direct and indirect interactions.

Another intriguing observation was that *A. muciniphila* gavage specifically inhibited STZ‐induced muscle atrophy without affecting the adipose masses. Abnormalities of skeletal muscles from individuals with T1DM, including altered protein synthesis and degradation, impaired glycolysis, mitochondrial dysfunction and ultrastructure changes [17, 18]. Not only the muscle mass and cross‐section areas, but also the metabolic‐related signaling pathways were increased by *A. muciniphila* gavage. These observations were quite different from those of obese and T2DM studies, in which *A. muciniphila* exterts antiobese and metabolic beneficial effects without changing the muscle mass and function [5, 19]. Since T1DM is different from the in obese and T2DM, especially considering the hyperinsulinemia, thus the antiatrophy ability of *A. muciniphila* may be dependent on the recovery of insulin signaling in muscle, evidenced by increased expression levels of *Igf2r* and *Igf2bp1*.

## CONCLUSION

In conclusion, we reported a previously unrevealed function of *A. muciniphila* gavage in protecting mice from STZ‐induced hyperglycemia and atrophy by promoting intestinal IGF2 secretion. Mice surviving after 5 months of STZ injection had reduced blood glucose levels and recovered gut microbiota with increased *A. muciniphila* proportion. Gavage of heat‐killed *A. muciniphila* increased the diversity of gut microbiota and elevated immune and metabolic signaling pathways in the intestine. Mechanistically, *A. muciniphila* treatment promoted the secretion of IGF2 from which subsequently activated IGF2 signaling in skeletal muscle. Our results suggest that the administration of heat‐killed *A. muciniphila* could be a potential therapeutic strategy for T1DM and its associated hyperglycemia.

## AUTHOR CONTRIBUTIONS

Yong Zhang and Zhihao Jia conceived the project. Zhihao Jia, Chi Zhang, and Zhihong Wang designed the experiments and prepared the manuscript. Chi Zhang, Zhihong Wang, Xu Liu, Xiangpeng Liu, and Zhengrong Yuan performed the experiments and analyzed the data. Tong Liu and Yu Feng provided key resources for the research. All authors have read the final manuscript and approved it for publication.

## CONFLICT OF INTEREST STATEMENT

The authors declare no conflict of interest.

## ETHICS STATEMENT

The ethics application (ZJ‐2021‐1) was approved by the CAM‐SU Animal Care and Use Committee.

## Supporting information


**Figure S1.** STZ injection altered SCFA metabolism of intestinal microbiota.
**Figure S2.** *A. muciniphila* administration protects mice from STZ‐induced weight loss and hyperglycemia.
**Figure S3.** *A. muciniphila* gavage promotes global metabolism.
**Figure S4.** Mice treated with *A. muciniphila* had enlarged muscle.
**Figure S5.** *A. muciniphila* administration promotes muscle metabolism.
**Figure S6.** *A. muciniphila* gavage reshapes bacteria community.
**Figure S7.** *A. muciniphila* gavage reshapes intestinal gene expression of STZ‐induced T1D mice.
**Figure S8.** IGF2 level is not changed in muscle after *A. muciniphila* administration.

**Table S1.** Bacterial community structures of mice at different times after STZ injection.
**Table S2.** Taxon of bacteria of mice at different times after STZ injection.
**Table S3.** RNA‐seq data of TA muscle from saline and AKK groups after STZ injection.
**Table S4.** Bacterial community structures at phylum level from mice in saline and AKK groups.
**Table S5.** RNA‐seq data of intestine from saline and AKK groups after STZ injection.
**Table S6.** RNA‐seq data of TA muscle from intestine of mice in saline, saline + STZ and AKK + STZ groups.

## Data Availability

All the sequencing data have been deposited in the Geno me Sequence Archive (GSA, https://ngdccncb.ac.cn/gsa with accession number: CRA018353 and attached in supplementary tables. Supplementary materials (methods, figures, tables, scripts, graphical abstract, slides, videos, Chinese translated version and updated materials) may be found in the online DOI or iMeta Science http://www.imeta.science/.
