# Supplementary material for: Akkermansia muciniphila administration ameliorates streptozotocin‐induced hyperglycemia and muscle atrophy by promoting IGF2 secretion from mouse intestine
Source: Imeta. 2024 Oct 1;3(5):e237. doi: 10.1002/imt2.237 (PMC11487547; doi:10.1002/imt2.237)
Supplement: Supplementary file 1 — Figure S1. STZ injection altered SCFA metabolism of intestinal microbiota. Figure S2. A. muciniphila administration protects mice from STZ‐induced weight loss and hyperglycemia. Figure S3. A. muciniphila gavage promotes global metabolism. Figure S4. Mice treated with A. muciniphila had enlarged muscle. Figure S5. A. muciniphila administration promotes muscle metabolism. Figure S6. A. muciniphila gavage reshapes bacteria community. Figure S7. A. muciniphila gavage reshapes intestinal gene expression of STZ‐induced T1D mice. Figure S8. IGF2 level is not changed in muscle after A. muciniphila administration. [file IMT2-3-e237-s001.docx]

**Supporting information to *Akkermansia muciniphila* administration ameliorates streptozotocin-induced hyperglycemia and muscle atrophy by promoting IGF2 secretion from mouse intestine**

Chi Zhang^1, 2, #^, Zhihong Wang^1, 2, #^, Xu Liu^1, 2^, Xiangpeng Liu^1^, Tong Liu^3^, Yu Feng^4^, Zhengrong Yuan^5^, Zhihao Jia^1,^ *, Yong Zhang^1, 2,^ *

^1^Cambridge-Suda Genomic Resource Center, Suzhou Medical College, Soochow University, Suzhou 215000, China.

^2^Jiangsu Key Laboratory of Neuropsychiatric Diseases Research, Soochow University, Suzhou 215000, China.

^3^Institute of Pain Medicine and Special Environmental Medicine, Nantong University, Nantong, 226019, China.

^4^Department of Endocrinology, The Second Affiliated Hospital of Soochow University, Suzhou 215000, China.

^5^College of Biological Sciences and Technology, Beijing Forestry University, Beijing 100083, China

^#^These authors contributed equally: Chi Zhang, Zhihong Wang

*Correspondence: [yong.zhang@suda.edu.cn](mailto:yong.zhang@suda.edu.cn) (Yong Zhang) [zhjia@suda.edu.cn](mailto:zhjia@suda.edu.cn) (Zhihao Jia)

**METHODS**

**Animal care**

Experimental mice used in this study all were in a C57BL/6N background and were housed in the animal facility of CAM-SU (Suzhou, China) with free access to acidified water and standard rodent chow food (radiated and autoclaved). All animal care and experimental use were performed according to protocols approved by the CAM-SU Animal Care and Use Committee. Animals were maintained with a 12h light/dark cycle, food, and water were available ad libitum, and the temperature (22 ± 2 °C) and humidity (40%–60%) were maintained. This animal study was performed following the ARRIVE guidelines.

**STZ injection, sample collection**

Single intraperitoneal injection of streptozotocin (STZ) was applied to induce Type 1 Diabetes Mellitus (T1DM) in mice. In brief, mice were fasting overnight before receiving an injection of freshly dissolved STZ (Sigma-Aldrich, St. Louis, MO) in citrate buffer with a dose of 100mg/kg. Mice from the control group were treated with an injection of equal volume of citrate buffer. The successful induced T1DM mice was confirmed by random blood glucose levels over 16.7 mmol/L (300 mg/dL) from the tail vein measured by a glucometer (Accu-Chek Active; Roche, Switzerland).

**16s rRNA sequencing**

DNA was extracted from mouse feces with the E.Z.N.A Soil DNA kit (OMEGA, USA) according to the manufacturer’s protocol. DNA concentration and purity were checked with a Nanodrop ND-2000 spectrometer (Thermo, USA). V4 region of 16S rRNA gene was amplified with a barcoded universal bacterial 515F and 806R (GTGYCAGCMGCCGCGGTAA, GGACTACNVGGGTWTCTAA). To minimize PCR reaction bias, each sample was amplified in triplicate with the following reaction conditions: 25 cycles of denaturation at 95 °C for 30 s, annealing at 55 °C for 30 s and extension at 72 °C for 45 s, with a final extension at 72 °C for 10 min. Equimolar amounts of PCR products assuming that amplicons of the same size has a similar molar mass from each sample were combined in a single tube for high-throughput sequencing on an Illumina MiSeq platform (Illumina, San Diego, USA) in Biomarker Technologies Corporation (Beijing, China) which produced paired-ended reads.

**Metagenome sequencing**

The microbial genomic DNA samples from mice cecal contents collected at different times post STZ injection were extracted and sequenced on the illumina HiseqXten/Novaseq/MGI2000 System by Aenta Life Sciences. Base calling on the original sequencing results was performed by bcl2fastq (v2.17.1.14) and preliminary quality analysis was conducted to obtain the raw data of sequencing samples (Pass Filter Data, PF), which were stored in FASTQ (abbreviated as fq) file format. Then the second-generation sequencing data quality statistical software cutadapt (v1.9.1) was used to remove splices and low-quality sequences from the PF and obtain Clean Data for subsequent information analysis. If there was host contamination in the sample, use BWA software (v0.7.12) to compare with the host genome and filter out reads that may originate from the host. Based on the optimized Clean Data, use MEGAHIT (v1.1.3) software for the data assembly.

Prodigal (v3.02) software was used for coding gene prediction, then integrated the gene sequences of all samples, and further reduced redundancy through sequence clustering software MMseq2. SoapAligner (version 2.21) alignment software was applied to align the preprocessed clean reads to the constructed non redundant gene set Unigene sequence and obtain the number of reads of every Unigenes in each sample. Sequences of the Unigenes were then searched by Diamond (version v0.8.15.77) and aligned with the NR database to obtain taxonomic annotation information.

***A. muciniphila* culture and gavage**

*Akkermansia muciniphila* (*A. muciniphila*) MucT (ATCC BAA-835) was grown on brain heart infusion broth (BHI) plate supplemented with mucin in an anaerobic culture bag (bio-72871, Biobw, China) with CO_2_ producing bag (bio-72870, Biobw, China) and O_2_ sensor (bio-56236, Biobw, China). After 3-day of culture, bacterial colonies were scraped and washed with sterile saline, finally re-suspended in sterile saline with a concentration of OD_600_ = 1. The bacterial suspension was then autoclaved at 121 °C for 20 min to generate heat-killed *A. muciniphila* and stored at -80 °C until use.

Sodium acetate and sodium propionate dissolved in 0.9% saline with a final dose of 500mg/kg, this dosage of acetate was similar to that used to suppress lipolysis in previous studies and was shown to be well tolerated [1]. Oral gavage of *A*. *muciniphila* (2 × 10^8^ colony-forming units/300 µl) [2,3], sodium acetate and sodium propionate were performed every other day through curved stainless steel feeding needles (12G, 55 mm). Only heat-killed *A. muciniphila* was used to treat the mice in this study.

**Body composition measurement**

Minispec LF50 body composition analyzer located in Small Animal Facility of CAM-SU was applied to measure the total body fat and lean mass of unanesthetized live animals. In brief, mice were placed in a specially sized, clear plastic holder without sedation or anesthesia. The holder was then inserted into a tubular space located in the side of the Minispec LF50 system. The mice were forced to not move in the holder to guarantee the accuracy of results. Each scan took approximately 80 seconds.

**Indirect calorimetry and body composition measurement**

Continuous day and night Oxygen consumption (VO_2_), carbon dioxide production (VCO_2_), respiration exchange rate (RER) and energy expenditure (EE) of control and *A*. *muciniphila* treated mice were measured through an indirect calorimetry system (Oxymax, Columbus Instruments). Mice were placed in each chamber individually and had free access to both food and water in an environmental temperature of 24 °C and 12-h light (8AM-8PM)-dark (8PM-8AM) cycle. The experiments and data were conducted and recorded over 4 days. Average day and night data were the average of all data points throughout the 12-hour cycle.

**GTT and ITT**

For the glucose tolerance test (GTT), mice were fasted overnight before receiving an i.p. injection of 10 μL/g body weight D-glucose (200 mg/ml). The blood glucose concentrations were measured from tail vein blood drops through a glucometer (Accu-Chek Active; Roche, Switzerland) at 0, 15-, 30-, 60- and 120-min post injection. For the insulin tolerance test (ITT), after fasting for 6 h, mice were given an i.p. injection of 10 μL/g body Insulin (4 μg/ml). Blood glucose concentrations were measured from tail vein blood drops through a glucometer (Accu-Chek Active; Roche, Switzerland) at 0, 30-, 60-, 90- and 120-min post injection. Mice were single and randomly caged with blinded cage numbers in order throughout each test.

**H&E staining**

Dissected tissues were fixed in 4% paraformaldehyde dissolved in 0.01M phosphate buffer (pH 7.4), dehydrated with gradient ethanol and embedded in embedding agent, before cutting into 12 μm sections on a Leica microtome. The sections were stained with H&E using standard procedures (removing the dewaxing step). The thickness of the tibialis anterior muscle was quantified. The cross-section area of muscle fibers was quantified by Image J. Three muscle samples from three mice in each experimental group were used for quantification.

**Total RNA extraction and real-time PCR**

Total RNA was extracted by using TRIzol reagent (Invitrogen) according to the protocol by the manufacturer. Briefly, the mouse muscles and intestines were collected and added to precooled TRIzol (1 ml) according to the size of the tissue and homogenized for a total of 45–60s using a simple laboratory homogenizer. cDNAs were synthesized from total RNA using a reverse transcription kit (R232-01; Vazyme Biotech) following the supplier's instructions. Real-time polymerase chain reaction (PCR) was conducted by using SYBR Green PCR Master Mix (Q711-02; Vazyme Biotech) on an Opticon real-time PCR Detection System (Applied Biosystems). The relative fold of change in expression was calculated by using the 2(-Delta Delta C(t)) method after normalization to heat shock protein 90 (HSP90) expression. Primers were synthesized by Genewiz, and are shown as below:

| Primer | Sequence (5’to 3’) |
| --- | --- |
| *Igf2r*-F | GGGAAGCTGTTGACTCCAAAA |
| *Igf2r*-R | GCAGCCCATAGTGGTGTTGAA |
| *Hgf*-F | ATGTGGGGGACCAAACTTCTG |
| *Hgf*-R | GGATGGCGACATGAAGCAG |
| *Igf2bp1*-F | CTCAGTCCCCAAAAAACAAAGG |
| *Igf2bp1*-R | TCGGAGCTGAGGTGGAATAT |
| *Bfgf*-F | GCGACCCACACGTCAAACTA |
| *Bfgf*-R | TCCCTTGATAGACACAACTCCTC |
| *Atrogin1*-F | CAGCTTCGTGAGCGACCTC |
| *Atrogin1*-R | GGCAGTCGAGAAGTCCAGTC |
| *Trim63*-F | GTGTGAGGTGCCTACTTGCTC |
| *Trim63*-R | GCTCAGTCTTCTGTCCTTGGA |
| *Mstn*-F | AGTGGATCTAAATGAGGGCAGT |
| *Mstn*-R | GTTTCCAGGCGCAGCTTAC |
| *Actin*-F | CACACCTTCTACAATGAGCTGC |
| *Actin*-R | GGCATAGAGGTCTTTACGGATG |

**Protein extraction and western blot analysis**

Protein samples were collected by using RIPA lysates containing protease inhibitors and phosphatase inhibitors (Selleck). To measure the protein concentration, the bicinchoninic acid assay (BCA) method with a total protein assay kit (Thermo Fisher) was used. The Biofuraw™ Precast Gel (Tanon) was used to perform western blot, and the sample of each well was 10 μg, and then electrophoretically transferred onto polyvinylidene difluoride membrane (PVDF) (Millipore), 90V voltages for 120 min. The membranes were blocked with 5% nonfat milk in Tris-buffered saline containing 0.1% Tween-20 for 1h at 37 °C and then incubated with primary antibodies overnight at 4 °C. The membranes were washed three times with Tris-buffered saline Tween-20 (TBST), and then incubated with secondary anti- bodies (Jackson) for 1h at 37 °C and then quantitative western blot images were recorded with a fully automated chemiluminescence image analysis system (Clinx). The protein bands were measured and statistically analyzed by the software Image J and averaged with the control group for normalization. The following primary antibodies were used: rabbit anti-HSP90 (1:1000; Cat#: 60318-1-Ig; Proteintech), rabbit anti-ATGL (1:1000; Cat#:2439S; CST), rabbit anti-IGF2 (1:1000; Cat#: 40941; CST), rabbit anti-MSTN (1:1000; Cat#:19142-1-AP; Proteintech), mouse anti-Total OXPHOS (1:1000; Cat#: ab110413; abcam). The following secondary antibody was used: donkey anti-rabbit IgG (H+L) (1:10000; Cat#:711-035-152; Jackson), goat anti-mouse (1:10000; 115-035-003; Jackson).

**ELISA**

Mouse blood samples from control and AKK groups were collected through retro-orbital blood and serum was then separated by centrifugation at 5000 rpm for 15 min at 4 °C and stored at -80 °C until analysis. Serum IGF2 concentrations were measured in all study subjects using specific ELISA kits (Elabscience, Elabscience Biotechnology Co., Ltd, China), according to the manufacturer’s instructions. All samples were tested at least twice for confirmation.

**Transcriptome sequencing**

RNA was isolated from mouse muscles and intestines using TRIzol reagent (15596026, Invitrogen), following the manufacturer’s instructions, and sent to Biomarker Technologies Corporation for library preparation and sequencing. The FPKM value was adopted to calculate gene expression. For differential expression analysis, *p* ≤ 0.01, and |Log2 (fold change) |≥ 1.5 were set as inclusion criteria. KEGG analysis was performed with a public online database.

**Statistical analysis**

Data were analyzed using GraphPad Prism 8.0 (GraphPad Software, CA, USA). All analyses between two-groups were conducted with Student’s *t* test (two-tailed), comparisons of metabolic chamber results were conducted by two-way ANOVA and ANCOVA analysis. All experimental data are presented as mean ± SEM (standard error of mean). Comparisons with *p* values < 0.05 were considered statistically significant.

**REFERENCES**

1. Frost, Gary, Michelle L Sleeth, Meliz Sahuri-Arisoylu, Blanca Lizarbe, Sebastian Cerdan, Leigh Brody, Jelena Anastasovska, Samar Ghourab, Mohammed Hankir, Shuai Zhang. 2014. “The short-chain fatty acid acetate reduces appetite via a central homeostatic mechanism.” *Nature communications* 5: 3611. doi: 10.1038/ncomms4611.

2. Zhang, Jing, Yueqiong Ni, Lingling Qian, Qichen Fang, Tingting Zheng, Mingliang Zhang, Qiongmei Gao, Ying Zhang, Jiacheng Ni, Xuhong Hou. 2021. “Decreased abundance of *Akkermansia muciniphila* leads to the impairment of insulin secretion and glucose homeostasis in lean type 2 diabetes.” *Advanced Science* 8: 2100536. doi: 10.1002/advs.202100536.

3. Lawenius, Lina, Julia M Scheffler, Karin L Gustafsson, Petra Henning, Karin H Nilsson, Hannah Colldén, Ulrika Islander, et al. 2020. “Pasteurized *Akkermansia muciniphila* protects from fat mass gain but not from bone loss.” *American journal of physiology-endocrinology metabolism* 318: E480-E491. doi: 10.1152/ajpendo.00425.2019.


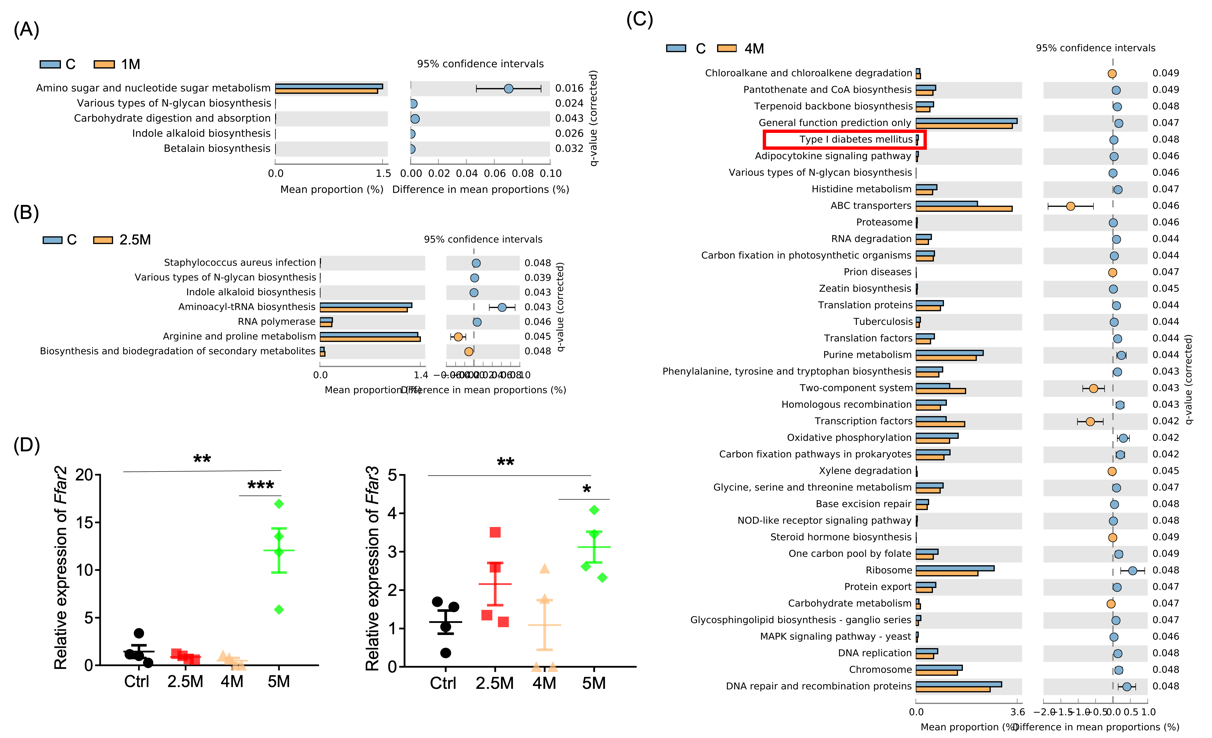


**Figure S1 STZ injection altered SFCA metabolism of intestinal microbiota.**

(A-C) KEGG pathway analysis of bacterial communities after streptozotocin (STZ) injection, *n* = 6. (D) qRT-PCR detection of *Ffar2* and *Ffar3* at 2.5-month 4-month and 5-month post STZ injection. Data represent mean ± SEM (*t*-test: **p* < 0.05, ***p* < 0.01, ****p* < 0.001).


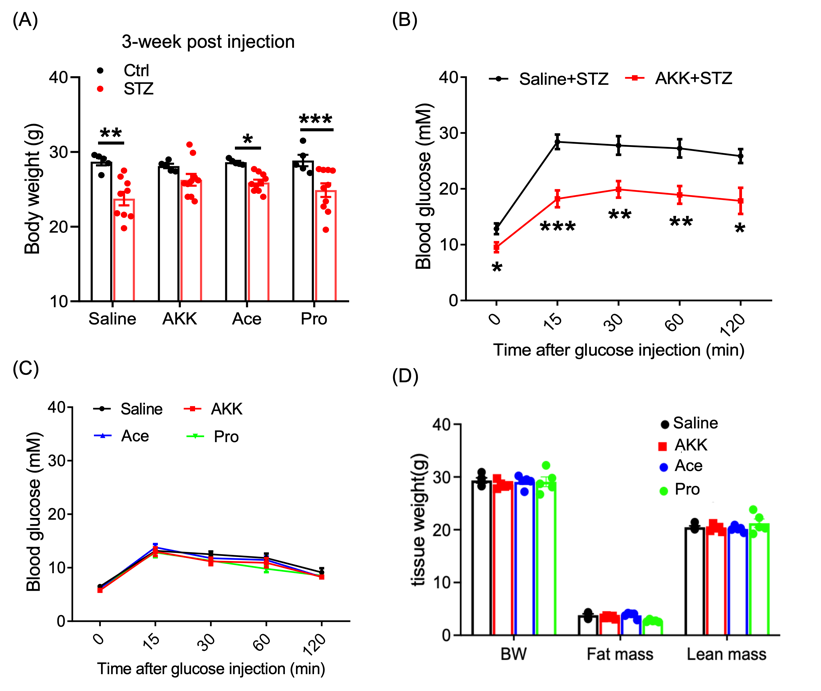


**Figure S2** ***A. muciniphila* administration protects mice from STZ-induced weight loss and hyperglycemia.**

(A) Body weight of mice received gavage of Saline, *Akkermansia muciniphila* (*A. muciniphila*), sodium acetate (Ace) and sodium propionate (Pro) after 3- of STZ induced Type 1 Diabetes Mellitus (T1DM), *n* = 5 and 10 of mice with/without STZ injection. (B) Blood glucose levels during glucose tolerance test (GTT) on mice after STZ injection, *n* = 7. (C) Blood glucose levels during glucose tolerance test (GTT) on mice without STZ injection. (D) Area under curve (AUC) calculated from GTT. Data represent mean ± SEM (*t*-test: **p* < 0.05, ***p* < 0.01, ****p* < 0.001).

**
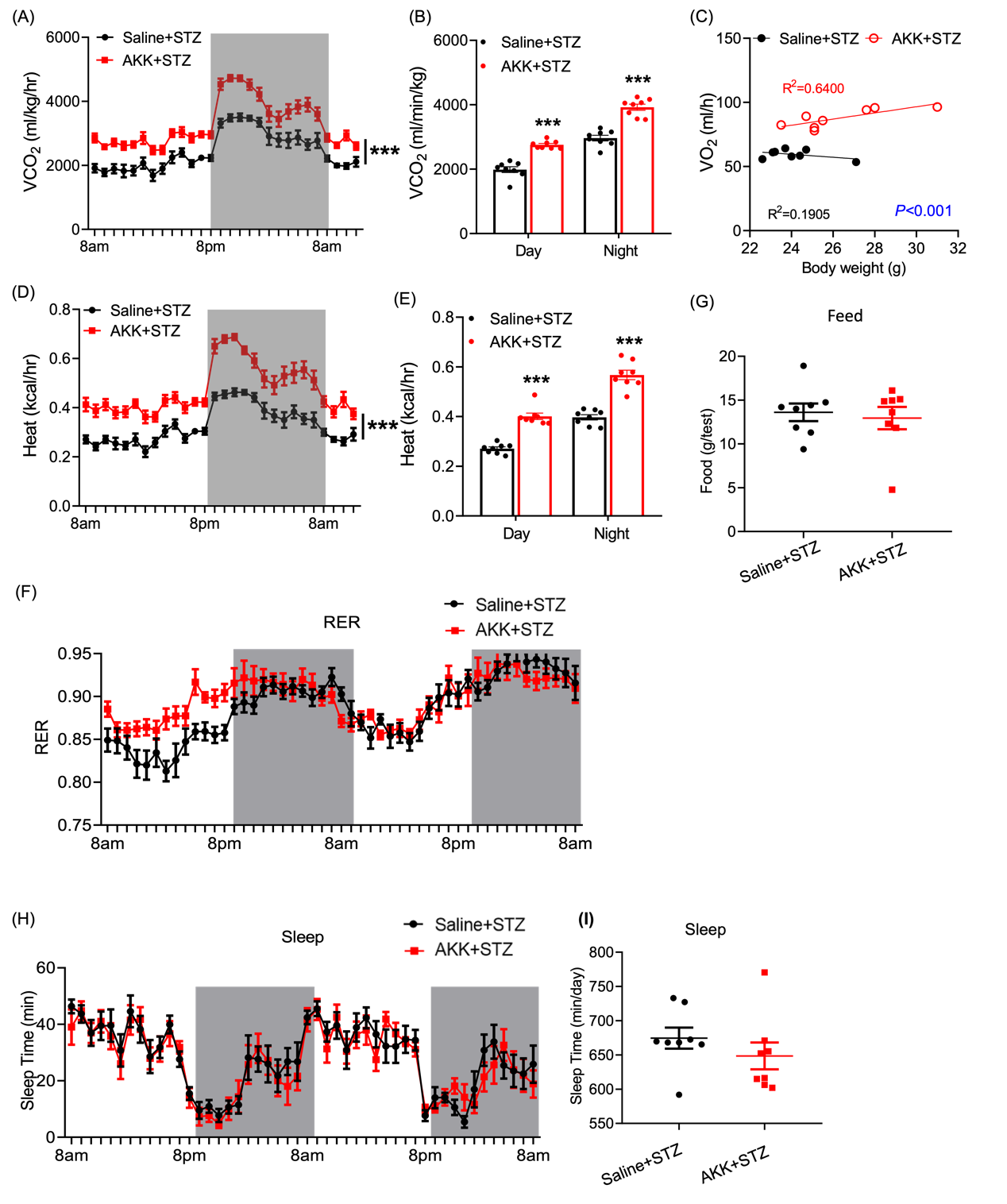
**

**Figure S3** ***A. muciniphila* gavage promotes global metabolism.** (A-C) CO_2_ production (A), average day and night CO_2_ production (B) and correlation between CO_2_ production and body weight (C) from AKK and Saline groups, *n* = 8. (D, E) Heat production (D) and average day and night heat production (E) from AKK and Saline groups, *n* = 8. (F-I) Average food intake (E), RER (F), sleep time (G) and average sleep time (H) of the mice from AKK and Saline groups, *n* = 8. Data represent mean ± SEM (*t*-test: ****p* < 0.001).

**
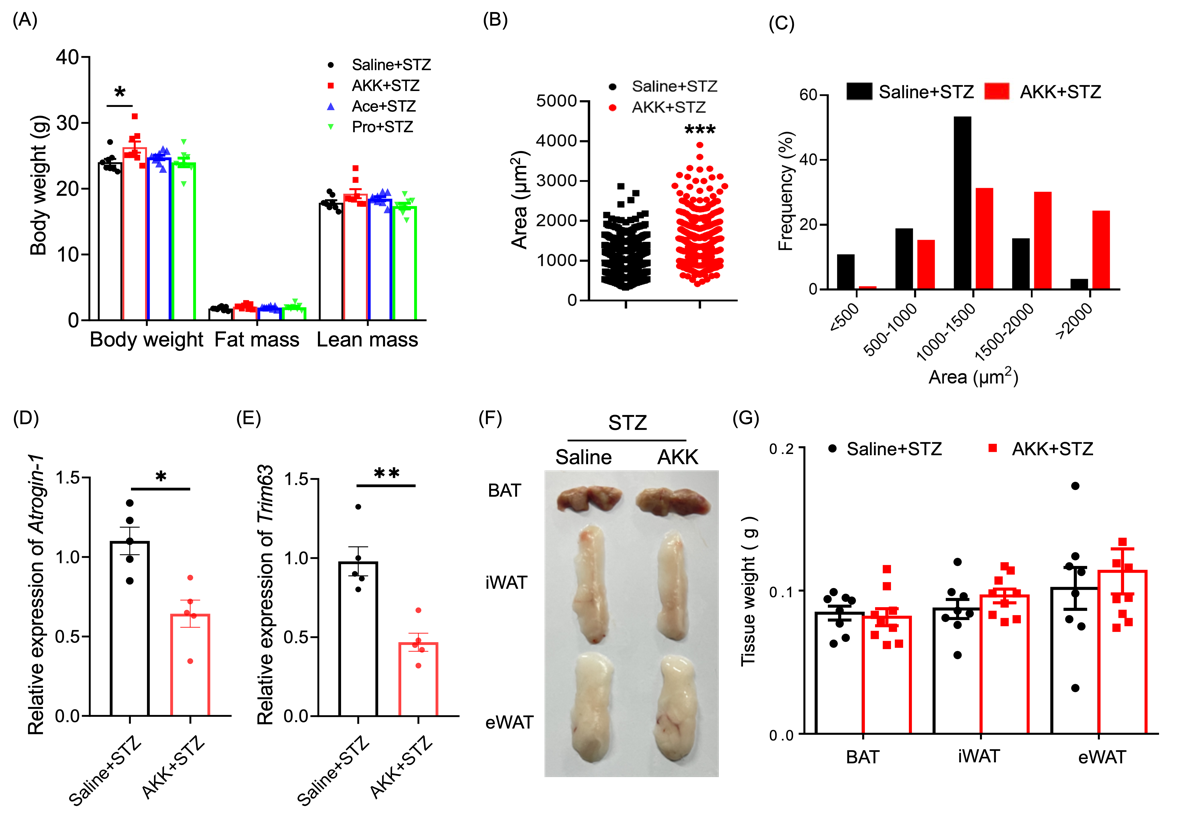
**

**Figure S4** **Mice treated with *A. muciniphila* had enlarged muscle.** (A) Body composition analysis of mice received gavage of Saline, AKK, Ace and Pro after STZ injection measured by MRI, *n* = 8. (B, C) Average (B) and distribution (C) of muscle fiber cross-section area from Saline and AKK groups after STZ injection. (D, E) Relative mRNA levels of *Atrogin-1* (D) and *Trim63* (E) in tibialis anterior (TA) muscles from Saline and AKK groups after STZ injection. (F, G) Representative images (F) and weights (G) of brown adipose tissue (BAT), inguinal white adipose tissue (iWAT) and epididymal WAT (eWAT) isolated from mice received gavage of Saline and AKK after STZ induced T1D. Data represent mean ± SEM (*t*-test: **p* < 0.05, ***p* < 0.01, ****p* < 0.001).

**
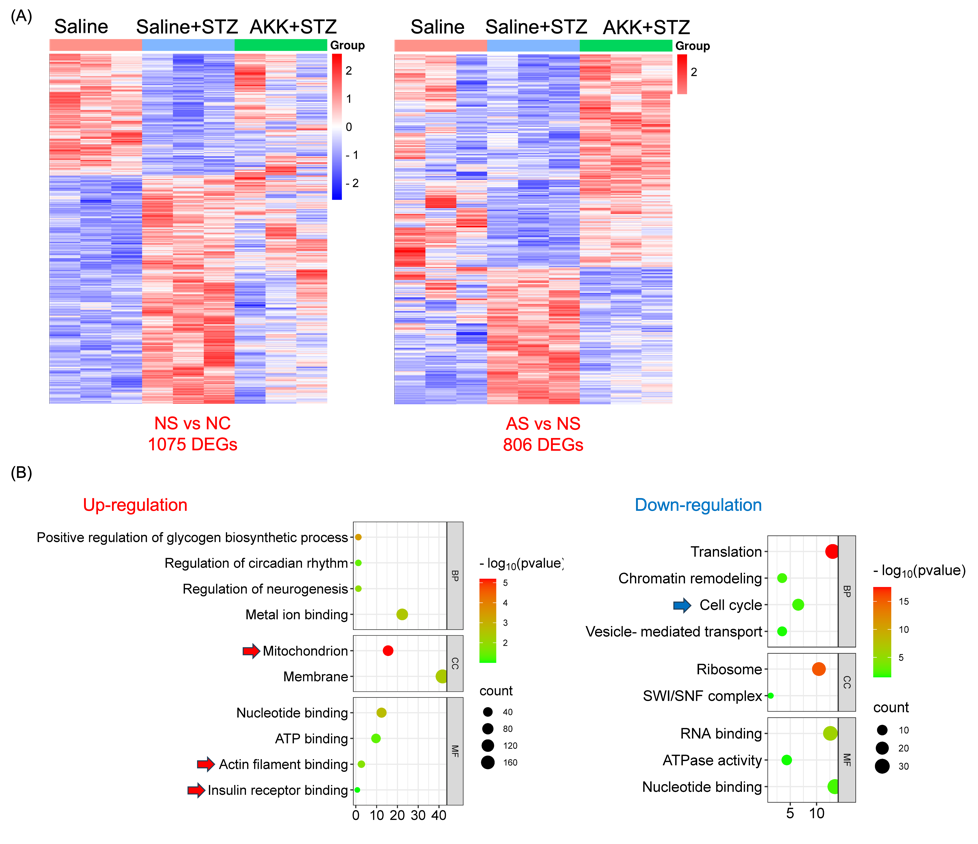
**

**Figure S5** ***A. muciniphila* administration promotes muscle metabolism.** (A) Heatmap of all differentially expressed genes (DEGs) Saline VS Control and AKK VS Saline after STZ injection, *n* = 3. (B) GO enrichment of upregulated and downregulated DEGs from TA muscles of AKK group compared to Saline group, *n* = 3.


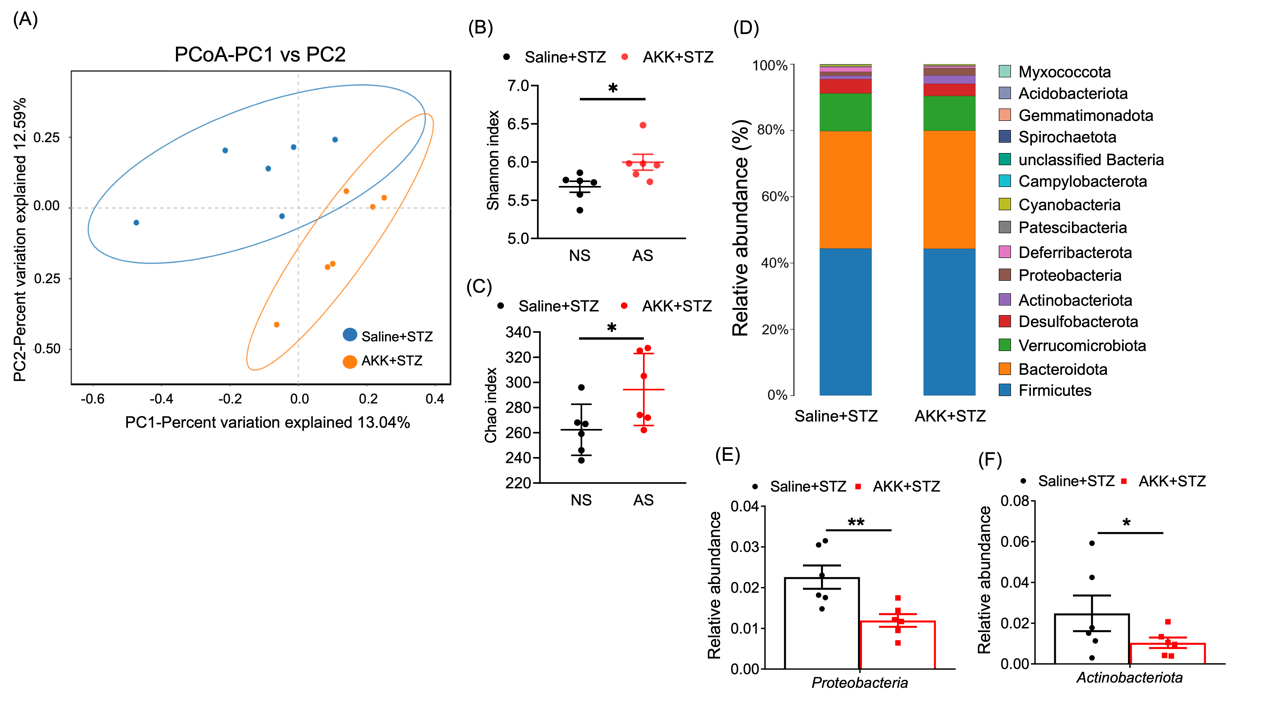


**Figure S6** ***A*. *muciniphila* gavage reshapes bacteria community of STZ-induced T1D mice.** (A) PCA analysis of 16s rRNA sequencing data of cecal contents from mice from AKK and Saline groups after STZ injection, *n* = 6. (B, C) Shannon (B) and Chao1 (C) indexes of 16s rRNA sequencing data, *n* = 6. (D) Bacterial community structure analysis at phyla level, *n* = 6. (E, F) Relative abundances of Proteobacteria (E) and Actinobacteriota (F) from AKK and Saline groups after STZ injection, *n* = 6. Data represent mean ± SEM (*t*-test: **p* < 0.05, ***p* < 0.01).


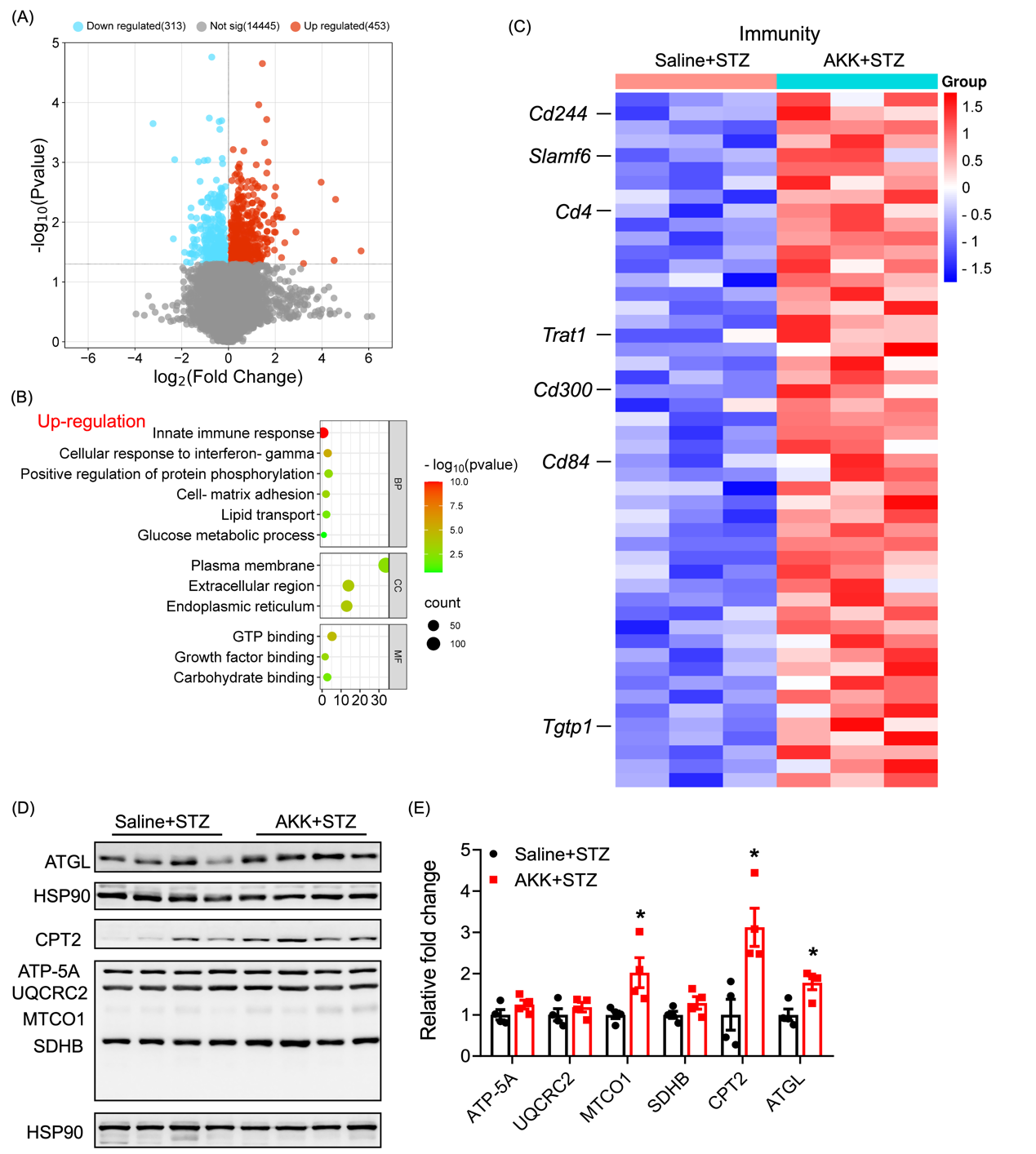


**Figure S7** ***A*. *muciniphila* gavage reshapes intestinal gene expression of STZ-induced T1D mice.** (A) Volcano plot of showing the Log_2_ fold change and -Log_10_*P* of intestinal genes by RNA-sequencing, red dots represented significant upregulated DEGs while blue dots represented significant downregulated DEGs, *n* = 3. (B) GO enrichment of upregulated DEGs from intestine of AKK group compared to Saline group, *n* = 3. (C) Heatmap of all DEGs in immunity pathway. (D, E) Western blot (D) and calculation (E) of CPT2, ATGL and mitochondrial complex proteins from intestine of AKK and Saline groups after STZ injection, *n* = 4. Data represent mean ± SEM (*t*-test: **p* < 0.05).


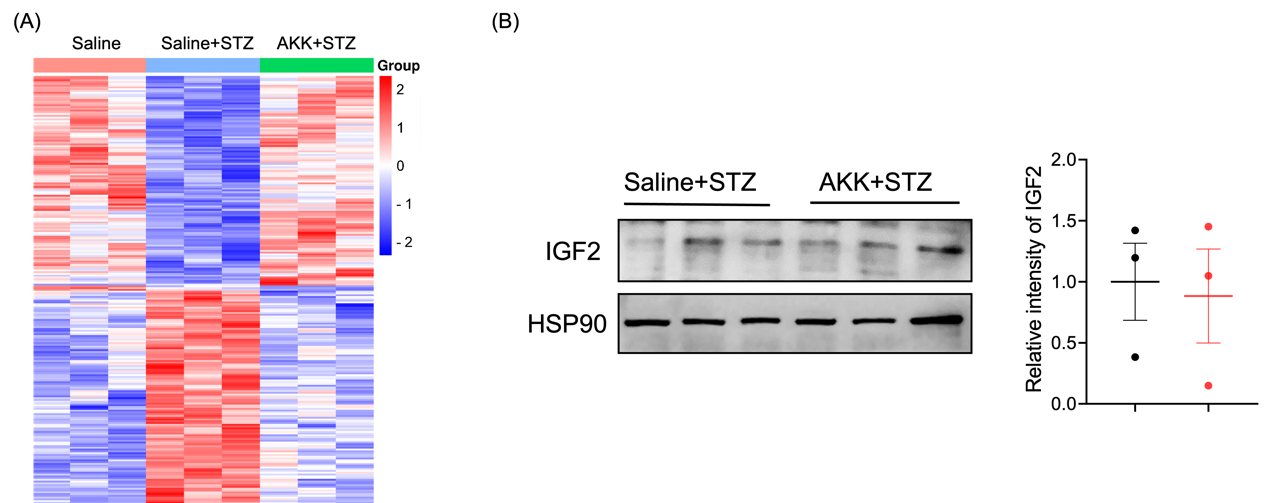


**Figure S8 IGF2 level is not changed in muscle after *A. muciniphila* administration.** (A) Heatmap of all overlapping DEGs in three groups. (B) Western blot and calculation of IGF2 protein from TA muscles of AKK and Saline groups after STZ injection.
